# 56. Long-Term Cardiovascular Outcomes After Drug-Related vs Non-Drug-Related Infective Endocarditis

**DOI:** 10.1093/ofid/ofab466.056

**Published:** 2021-12-04

**Authors:** Brandon Muncan, Aikaterini Papamanoli, Hal A Skopicki, Andreas Kalogeropoulos

**Affiliations:** Stony Brook University Hospital, Stony Brook, NY

## Abstract

**Background:**

Drug use-related infective endocarditis (IE) has nearly doubled in the past two decades in the United States, largely due to the current opioid crisis. Although there are robust data on surgical outcomes for people who use drugs (PWUD) vs. non-PWUD patients after an initial encounter for IE, long-term comparative data on post-IE outcomes are relatively sparse.

**Methods:**

Using data from the TriNetX electronic health records network, we identified (1) a cohort of patients 16 to 64 years old who had a first encounter for IE (captured with ICD-10 codes I33, I38, or I39) and history of drug use (captured with ICD-10 codes F11, F13-F16, F18, F19, O99.32, or T40) preceding the IE episode and (2) a propensity score-matched cohort of patients age 16-64 who had a first episode of IE and no documented drug use. We compared the post-IE incidence of (1) mortality; (2) ischemic stroke; (3) intracranial hemorrhage; (4) myocardial infarction; (5) heart failure; and (6) sudden cardiac death (cardiac arrest or ventricular fibrillation or tachycardia) between the 2 cohorts over a 5-year follow up period. We matched the cohorts for demographic data and clinically relevant medical history. We used Kaplan-Meier estimates and Cox models to compare incidence.

**Results:**

We identified 6,578 PWUD patients and 6,578 matched non-PWUD patients 16-64 years old with a first episode of IE. The baseline characteristics are summarized in **Table 1**. Standardized mean differences of characteristics were generally < 0.1, indicating adequate matching. The 5-year Kaplan-Meier rates of outcomes of interest are summarized in **Table 2**. Mortality did not differ between cohorts. However, the incidence of ischemic stroke and intracranial hemorrhage was consistently higher among PWUD throughout the 5-year follow-up. Rates of myocardial infarction were also higher among PWUD; however, the difference was more pronounced later during follow-up. Rates of heart failure and sudden cardiac death did not differ.

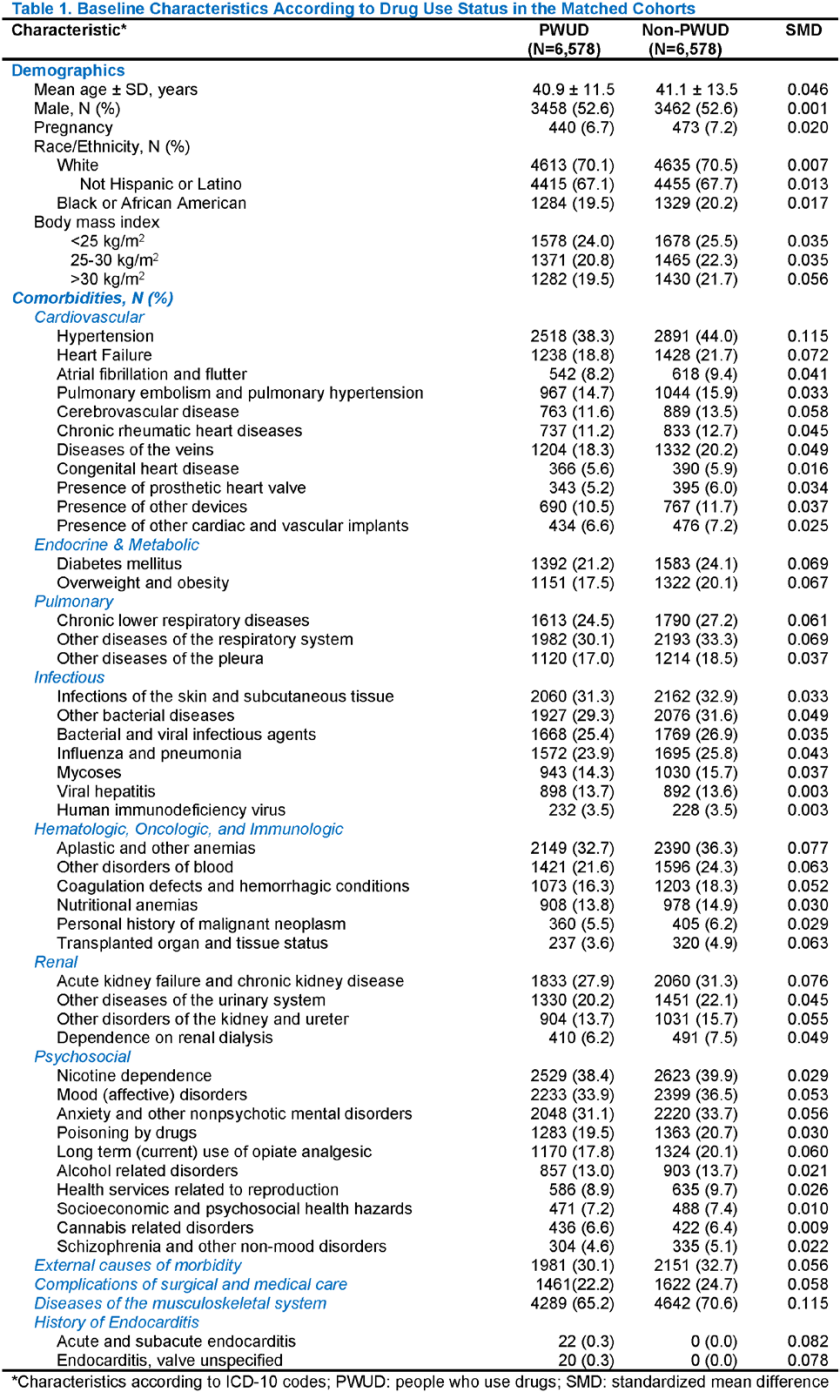

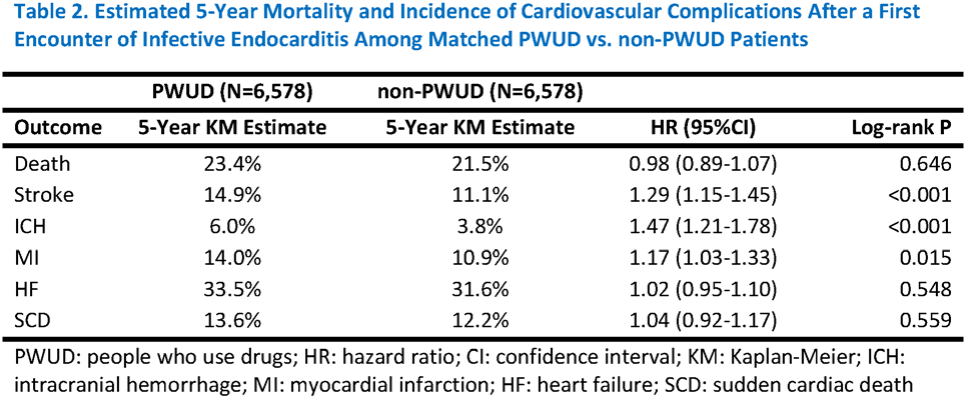

**Conclusion:**

Cardiovascular events after IE were common among both PWUD and non-PWUD patients over a 5-year follow-up period. However, rates of ischemic and hemorrhagic stroke were consistently higher among PWUD. Further investigation is needed to elucidate the sources of elevated stroke risk among PWUD and identify targets for intervention.

**Disclosures:**

**All Authors**: No reported disclosures

